# FGFC1 overcomes Ara-C resistance in acute myeloid leukemia by inducing apoptosis and pyroptosis

**DOI:** 10.3389/fphar.2025.1584376

**Published:** 2025-08-14

**Authors:** Xiaohui Hu, Zhijian Li, Rui Zhou, Bing Zhang, Ruoxian Wang, Tongtong Li, Jiangcheng Chang, Wenhui Wu, Ning Liu

**Affiliations:** ^1^ International Research Centre for Food and Health, College of Food Science and Technology, Shanghai Ocean University, Shanghai, China; ^2^ Marine Biomedical Science and Technology Innovation Platform of Lin-gang Special Area, Shanghai, China; ^3^ Department of Chemistry, College of Food Science and Technology, Shanghai Ocean University, Shanghai, China; ^4^ Shanghai Engineering Research Center of Aquatic-Product Processing and Preservation, Shanghai, China

**Keywords:** acute myeloid leukemia (AML), cytarabine (Ara-C) resistance, FGFC1, apoptosis and pyroptosis, GSDME

## Abstract

Acute myeloid leukemia (AML) is a hematologic malignancy with a high mortality rate and poor prognosis, largely attributed to the emergence of chemotherapy resistance. Cytarabine (Ara-C), the cornerstone chemotherapeutic agent for AML, faces significant challenges due to the development of resistance, creating an urgent need for novel therapeutic strategies. Pyroptosis as a new form of programmed cell death has emerged as a potential therapeutic target in tumor treatment. However, its role in overcoming Ara-C resistance in AML by modulating pyroptosis remains unexplored. FGFC1 (Fungi fibrinolytic compound 1) a natural compound derived from *Stachybotrys longispora* FG216, has previously been shown to have high efficacy against erlotinib-resistant non-small cell lung cancer, yet its effects on AML are unknown. This study demonstrated that FGFC1 overcame Ara-C resistance in AML by inducing apoptosis and pyroptosis. Mechanistically, FGFC1 induced mitochondrial dysfunction and the accumulation of intracellular reactive oxygen species (ROS), leading to the release of cytochrome c (Cyto-C), which activated Caspase-3 and triggered both apoptosis and pyroptosis. This process was driven by inhibition of the PI3K/Akt/mTOR signaling cascade, ultimately suppressing the growth of AML Ara-C-resistant cells. These findings highlight the potential of FGFC1 to overcome Ara-C resistance in AML, providing a promising therapeutic strategy for drug-resistant AML and supporting the broader application of marine-derived small molecules in cancer therapy.

## 1 Introduction

Acute myeloid leukemia (AML) is a hematological malignancy characterized by clonal expansion of mutant progenitors with nearly one-third of all leukemia cases and the highest mortality rates among leukemia subtypes (Forsythe and Sandman, 2021; Venugopal and Sekeres, 2024). At present, the conventional treatment regimen is the “7 + 3” chemotherapy regimen consisting of Cytarabine (Ara-C) and Zoerythromycin, providing a high early remission rate, especially in younger patients (Liu, 2021). However, the emergence of acquired resistance and other adverse factors has resulted in a 5-year survival rate of AML that remains below 30% (De Kouchkovsky and Abdul-Hay, 2016). Thus, identifying strategies for overcoming Ara-C resistance is urgently necessary to improve clinical outcomes.

Pyroptosis is a form of programmed cell death described as a more intense form of cell death than apoptosis (Bertheloot et al., 2021). Under the influence of various inducing factors, the cleavage of Gasdermin (GSDM) proteins by CASP proteins triggers cell swelling, rupture of the plasma membrane, and release of intracellular contents (Yu et al., 2021). In tumors, the relationship between pyroptosis and cancer is more complex. Recently, studies have shown that pyroptosis is strongly associated with the response to chemotherapy in various tumors (Dai et al., 2023; Su et al., 2023). There is a significant correlation between elevated pyroptosis risk scores and both chemotherapy resistance and disease recurrence in AML (Zhang et al., 2024a). Notably, the combination of anagrelide and idarubicin can induce pyroptosis mediated by GSDME, which may serve as a potential therapeutic approach for AML with high PDE3A expression (Yang et al., 2024). Evidence suggests that GSDME plays a role in tumor chemoresistance. In pancreatic cancer, GSDME-dependent pyroptosis has been shown to promote resistance to several chemotherapeutic agents, including gemcitabine, irinotecan, 5-fluorouracil, paclitaxel, and cisplatin (Li et al., 2023). GSDME cleavage by Caspase 3 determines lobaplatin-induced pyroptosis in colon cancer cells (Cheng, 2023). However, the specific mechanisms by which pyroptosis contributes to Ara-C resistance in AML are not fully understood. Consequently, exploring the relationship and mechanism between pyroptosis and Ara-C resistance provides new therapeutic strategies for AML Ara-C resistance treatment.

Studies indicated that drugs induce the pyroptosis of tumor cells and increase their sensitivity to chemotherapy as an effective strategy for anti-tumor therapies (Jia et al., 2023). Natural active small molecules often target multiple processes in tumor cells, providing a synergistic therapeutic effect and reversing resistance in tumor therapy (Lu et al., 2020; Zhang et al., 2022). Marine Natural Products (MNPs) are considered a vital source of drug lead compounds due to their novel structures, low bio-toxicity, and high activity (Yang et al., 2023). Despite the ocean being a vast repository of biological resources, the advancement of MNPs has been still limited due to unclear mechanisms of action and ambiguous targets (Ren et al., 2021; Haque et al., 2022). FGFC1 (Fungi fibrinolytic compound 1), a marine bisindole alkaloid with the chemical formula C_51_H_68_N_2_O_10_, was isolated and identified from the marine microbial strain *Stachybotrys longispora* FG216. Previous studies by our group have shown that FGFC1 selectively inhibits erlotinib-resistant non-small cell lung cancer by inhibiting the NF-κB and PI3K/Akt/mTOR pathway-mediated ROS elevation (Feng et al., 2022; Yan et al., 2022). These findings suggest the potential of FGFC1 as a lead compound for future tumor-resistance treatments. However, the role of FGFC1 in AML, especially in terms of whether it can induce pyroptosis to overcome Ara-C resistance, has not been reported. Herein, we indicated that FGFC1 effectively overcomes AML Ara-C resistance through apoptosis and GSDME-dependent pyroptosis both *in vivo* and *in vitro*. Further mechanistic studies revealed that FGFC1 suppresses the activation of PI3K/Akt/mTOR pathway to result in mitochondrial dysfunction and ROS accumulation contributing to Cyto-C liberation, consequently leading to cleavage of GSDME by activating Caspase 3, and finally overcoming Ara-C resistance. Our research has identified a marine natural compound FGFC1 that obviously overcomes Ara-C resistance in AML, thereby offering a promising candidate for overcoming the risk of drug-resistant relapse and enhancing the therapeutic efficacy of AML. This finding not only highlights the potential of marine-derived small molecule compounds in the treatment of AML but also demonstrates that pyroptosis might be a valuable direction for Ara-C resistance treatment.

## 2 Materials and methods

### 2.1 Cell culture

The HL-60 and K562 cell lines were obtained from the American Type Culture Collection (ATCC). All cell lines were routinely tested to confirm that they were free of *Mycoplasma*. K562 and HL-60 cell lines were cultured in RPMI-1640 (Gibco, United States). All culture media were supplemented with 10% FBS with 100 U/mL penicillin and 100 μg/mL streptomycin, and cells were cultured at 37°C in 5% CO_2_. Cells were kept at low passages (3–5 passages) once obtained from vendors. Ara-C-resistant HL-60 (HL-60-R) and K562 (K562-R) cells were generated by culturing cells in a medium with a gradient of progressively increasing drug concentrations, reaching final concentrations of 1.5 μM for HL-60-R and 12 μM for K562-R. For all assays, drug-resistant cells were cultured for 48 h in a drug-free medium before use in experiments.

### 2.2 Chemical compounds

Ara-C (≥99% purity) was purchased from AbMole CO., Ltd. FGFC1 (≥98% purity) was presented to Prof. Wenhui Wu of Shanghai Ocean University.

### 2.3 Antibodies and reagents

Antibodies against the following proteins were used with source and dilution ratios indicated: GSDMB(CST, #76439, 1:1000); GSDMD (CST, #97558, 1:1000); GSDME (Abcam, # ab215191, 1:1000); Caspase 3 (CST, #9662, 1:1000); Cleaved-caspase 3 (Cl-caspase 3) (CST, #9661, 1:1000); Bcl-2 (CST, #4223, 1:1000); Bax (CST, #5023, 1:1000); Cyto-C (Beyotime, #AF2047, 1:1000); PI3K p85 (CST, #4292,1:1000), phospho-PI3K p85 (Tyr458)/p55 (Tyr199) (CST, #4228, 1:1000), Akt (CST, #9272, 1:1000), phospho-Akt (Ser473) (CST, #9271, 1:1000), mTOR (CST, #2983, 1:1000), and phospho-mTOR (Ser2448) (CST, #2971, 1:1000); β-actin (CST, #3700, 1:10000); Anti-rabbit lgG Fab2 (Sigma, #A0545, 1:10000); Anti-mouse lgG Fab2 (Sigma, #A4416, 1:10000). Phosphate-buffered saline (PBS) washing buffer, Fetal bovine serum (FBS), Trypsin-EDTA solution, and Penicillin-Streptomycin solution (PS) (100×) were all purchased from Gibco (Carlsbad, CA, United States). Cell Counting Kit-8 (CCK-8), RIPA lysis buffer, Bicinchoninic acid (BCA) Protein assay kit, Lactate dehydrogenase (LDH) Cytotoxicity Assay Kit, JC-1 assay kit, and ROS assay kit were purchased from Beyotime (Shanghai, China). The cocktail was obtained from MCE (Monmouth Junction, NJ, United States). Polyvinylidene fluoride (PVDF) membranes were purchased from Millipore (Billerica, MA, United States).

### 2.4 Cell viability assay

AML cells in the logarithmic growth phase with 70%–80% cell confluence were taken for centrifugation and resuspended in cell culture dishes using RPMI-1640 complete medium in preparation for cell inoculation. The inoculation volume of the 96-well plate was 5 × 10^3^ wells. The AML cells were incubated at 37°C for about 24 h, and were subjected to different concentrations of drug treatments, with DMSO as the control group, and three parallel groups were set up for each concentration of drugs. After 72 h of drug treatment, 10 μL of CCK-8 cell viability assay reagent was added to each well, and the absorbance (OD450 nm) value was detected by an enzyme marker after incubation at 37°C and avoiding light. The Combination Index (CI) used to assess the synergistic effects was calculated with CompuSyn software (ComboSyn, Inc.). The CI quantitatively defines the synergistic effect of a drug combination as follows: CI = 1 indicates an additive effect, CI < 1 indicates synergism, and CI > 1 indicates antagonism.

### 2.5 Animal studies

Selected 4–5-week NOD/SCID female nude mice were kept in SPF-grade chambers for 3–7 days at constant temperature (25°C) and relative humidity (65%) with a 12-h light/dark cycle. K562-R cells (5 × 10^6^) were inoculated subcutaneously in 6 ∼ 7-week-old mice. Every 2 days, the weight of the mice and the tumor volume were measured and recorded using a balance and vernier calipers and calculated using the standard formula: Volume = length × width^2^ × 0.5, with the tumor volume exceedingly approximately 100 mm^3^. The mice were randomly divided into 4 groups (6 mice per treatment group): blank group (2% DMSO dissolved in PBS, subcutaneous injection), FGFC1 (30 mg/kg/d, subcutaneous injection), Ara-C (30 mg/kg/d, subcutaneous injection), and FGFC1 + Ara-C (30 mg/kg/d + 30 mg/kg/d, subcutaneous injection) were administered continuously for 3 weeks. The mice were analyzed to determine whether they were in a state of near-death according to the principles of ethical review set forth by the Chinese National Standard for Animal Welfare and NC3Rs as a criterion for judging the humane endpoints of experimental animals. Mice were humanely euthanized prior to reaching the experimental endpoint if they exhibited any of the following:

a. 20% body weight loss within 72 h;
b. Tumor burden >15% of body weight or ulcerated tumors >2,000 mm^3^;
c. Inability to access food or water for more than 24 h;
d. Severe lethargy (no spontaneous movement for >30 s upon stimulation).

Euthanasia was performed using CO_2_ inhalation followed by cervical dislocation, in accordance with AVMA guidelines.Animal care and euthanasia protocols were approved by the Institutional Animal Care and Use Committee of Shanghai Ocean University (Shanghai, China) (Permit #SHOU-DW-2024-018).

### 2.6 Hematoxylin-eosin (HE) and immunohistochemical staining

HE and Immunohistochemical staining were performed by Shanghai RecordBio Co., Ltd. (Shanghai, China). Tumor sections were immunostained with specific anti-Ki67, anti-Cl-Caspase 3, anti-GSDME-N antibodies. The images were captured using a Zhiyue WS-10 Digital Panoramic Scanner and analyzed by using Image-Pro Plus 6.0.

### 2.7 Apoptosis assay

Cell apoptosis was analyzed by an Annexin V-FITC Apoptosis Detection Kit (Beyotime, Shanghai, China). After 48 h of DMSO, FGFC1, Ara-C, or FGFC1+Ara-C treatment, cells were harvested, washed twice with PBS, and resuspended in 1x binding buffer. Annexin V-FITC Apoptosis Detection Kit was used to stain 1 × 10^6^ cells, then incubated for 15 min in the dark. Cell apoptosis was detected by BD FACS Celesta flow cytometry (New York, United States). Apoptosis data were analyzed by FlowJo software.

### 2.8 Lactate dehydrogenase (LDH) release assay

AML Ara-C-resistant cells were seeded into 96-well plates. After 24 h, the specified concentrations of DMSO, FGFC1, Ara-C, or the combination of FGFC1+Ara-C were added to the wells, and the cells were treated for an additional 24 h. Following treatment, the cells were centrifuged, and 120 μL of the supernatant was transferred to a new 96-well plate. To each well, 60 μL of LDH working solution (containing lactic acid, INT solution, and enzyme solution) was added and mixed thoroughly. The plate was then incubated at room temperature, protected from light, for 30 min. Absorbance was measured at 490 nm using a microplate reader, and the data were analyzed using GraphPad Prism 8.0 software.

### 2.9 Statistical analyses

All the experimental data were analyzed by GraphPad Prism 8.0 (GraphPad Software Inc., San Diego, CA, United States). Results were expressed as mean values ±SD from at least 3 independent experiments. Comparisons between the two groups were performed by Student’s t-test. *p < 0.05 was considered as being significant (*p < 0.05, **p < 0.01, ***p < 0.001, ****p < 0.0001).

## 3 Results

### 3.1 FGFC1 significantly overcomes Ara-C resistance *in vitro*

**FIGURE 1:**
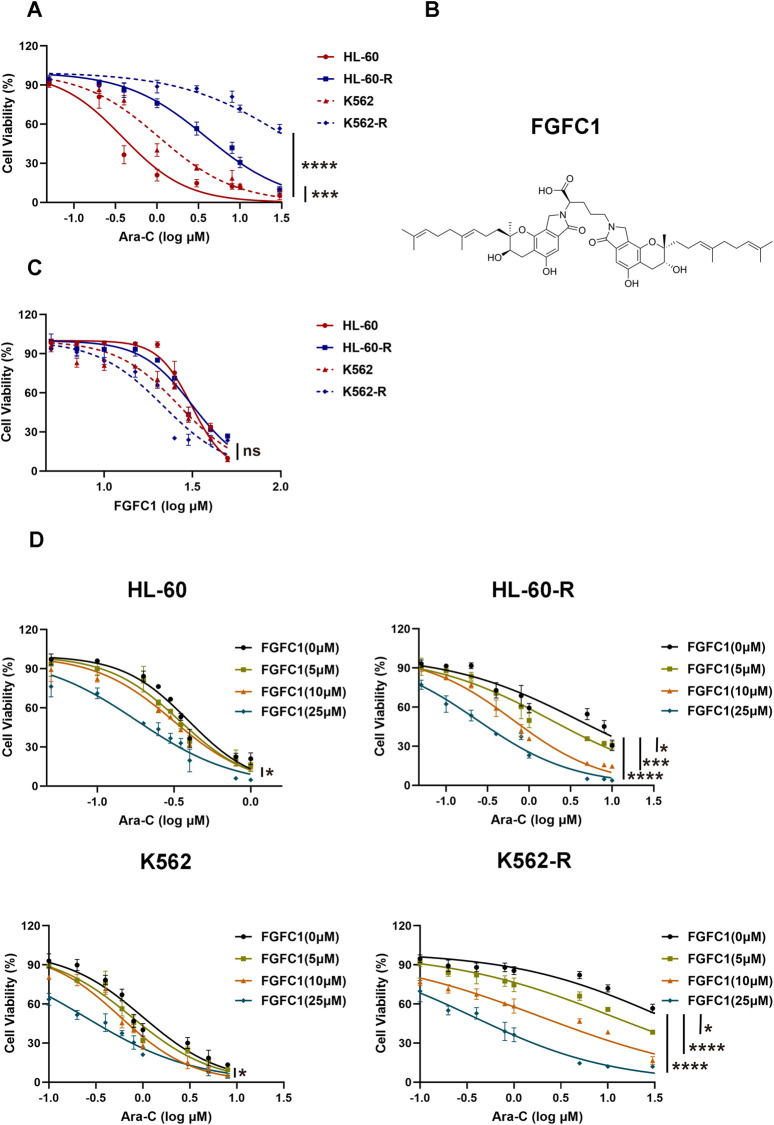
FGFC1 potentiates Ara-C-induced cytotoxicity in Ara-C-resistant AML cells *in vitro*. **(A)** HL-60, HL-60-R, K562, and K562-R cells were treated with different concentrations of Ara-C for 72 h. The activity was detected by CCK-8 and the IC_50_ value was calculated. **(B)** Chemical structure of FGFC1 (MW = 869.4947). **(C)** HL-60, HL-60-R, K562, and K562-R cells were treated with different concentrations of FGFC1 for 72 h. The activity was detected by CCK-8 and the IC_50_ value was calculated. **(D)** HL-60, HL-60-R, K562, and K562-R cells were treated with different concentrations of FGFC1 (0, 5, 10, and 25 μM) for 72 h. The activity was detected by CCK-8 and the IC_50_ values were calculated. All data were shown as mean ± SD, n = 3, Student’s t-test, *p < 0.05, **p < 0.01, ***p < 0.001, ****p < 0.0001.

To investigate the mechanism of Ara-C resistance in AML, *in vitro* Ara-C-resistant AML cell lines (K562-R and HL-60-R) were established. Drug-resistant cell lines were generated over a 6-month period using a stepwise Ara-C concentration gradient. Compared to the parent cells (K562 and HL-60), the drug-resistant cells (K562-R and HL-60-R) showed an over 10-fold increase in IC_50_ for Ara-C (Figure 1A; Table 1), which indicated that the Ara-C-resistant AML cell line was successfully established. The chemical structure of FGFC1 is shown in Figure 1B. However, for FGFC1, the IC_50_ values were quite close in the sensitive and resistant AML cells (Figure 1C; Table 1). Furthermore, we found that FGFC1 significantly increased cell sensitivity to Ara-C in a dose-dependent manner. In Ara-C-sensitive HL-60 and K562 cells, the Ara-C IC_50_ decreased from 0.38 μM and 1.06 μM to 0.18 μM and 0.24 μM, respectively, in the presence of FGFC1, whereas Ara-C-resistant HL-60-R and K562-R cells showed a decrease in the IC_50_ values from 3.93 μM and 36.94 μM to 0.24 μM and 0.38 μM. It can be seen that FGFC1 treatment significantly increased the sensitivity of HL-60 and K562 cells to Ara-C. Under FGFC1 10 μM treatment, the sensitivity rate of drug-resistant cells to Ara-C was about 5 and 10 times that of sensitive cells, respectively. This result indicated that FGFC1 had the effect of enhancing the efficacy of chemotherapeutic drugs on both sensitive and drug-resistant AML cells, and the impact on drug-resistant cells was significantly stronger than that on sensitive cells. The above results indicated that FGFC1 increased the sensitivity of AML cells to Ara-C (Figure 1D; Table 2). In addition, we explored the effect of FGFC1 combined with Ara-C on cell growth in Ara-C-resistant AML cells. As shown in Supplementary Figure S1, the combination of FGFC1 and Ara-C resulted in an obviously synergistic inhibition in Ara-C-resistant AML cells, with Combination Index (CI) value <1. These results proved that the ability of FGFC1 to increase the sensitivity of AML cells to Ara-C provides a new idea for the treatment of AML.

**TABLE 1:** The IC_50_ value of Ara-C and FGFC1 treating HL-60, HL-60-R, K562, and K562-R cells.

Drugs	IC_50_ (μM)			
	HL-60	HL-60-R	K562	K562-R
Ara-C	0.38 ± 0.01	3.39 ± 0.17^****^	1.06 ± 0.08	36.94 ± 0.88^****^
FGFC1	31.29 ± 0.51	31.75 ± 0.50	26.65 ± 0.29	21.59 ± 0.48

**TABLE 2:** The IC_50_ value and change of sensitivity of the HL-60, HL-60-R, K562, and K562-R cells to Ara-C after the treatment of different concentrations of FGFC1.

FGFC1 (μM)	IC_50_ (μM)				Sensitivity ratios [IC_50_ (-FGFC1)/IC_50_ (+FGFC1)]			
	HL-60	HL-60-R	K562	K562-R	HL-60	HL-60-R	K562	K562-R
0	0.38 ± 0.01	3.39 ± 0.17	1.06 ± 0.08	36.94 ± 0.88	1	1	1	1
5	0.34 ± 0.01	1.84 ± 0.04	0.76 ± 0.05	11.59 ± 0.61	1.12	2.14^*^	1.39	3.19^*^
10	0.30 ± 0.01	0.64 ± 0.05	0.58 ± 0.04	1.98 ± 0.04	1.27	6.14^***^	1.83	18.66^***^
25	0.18 ± 0.01	0.24 ± 0.01	0.24 ± 0.02	0.38 ± 0.01	2.11^*^	16.38^****^	4.42^**^	97.21^****^

“Sensitivity ratio”, which is the change in the sensitivity of cells to chemotherapeutic drugs after treatment with chemicals, was calculated as the ratio of the IC_50_ value of the drug to the IC_50_ value of the cells not treated with FGFC1 to the IC_50_ value of the cells treated with FGFC1.

### 3.2 FGFC1 overcomes AML Ara-C resistance *in vivo*

**FIGURE 2:**
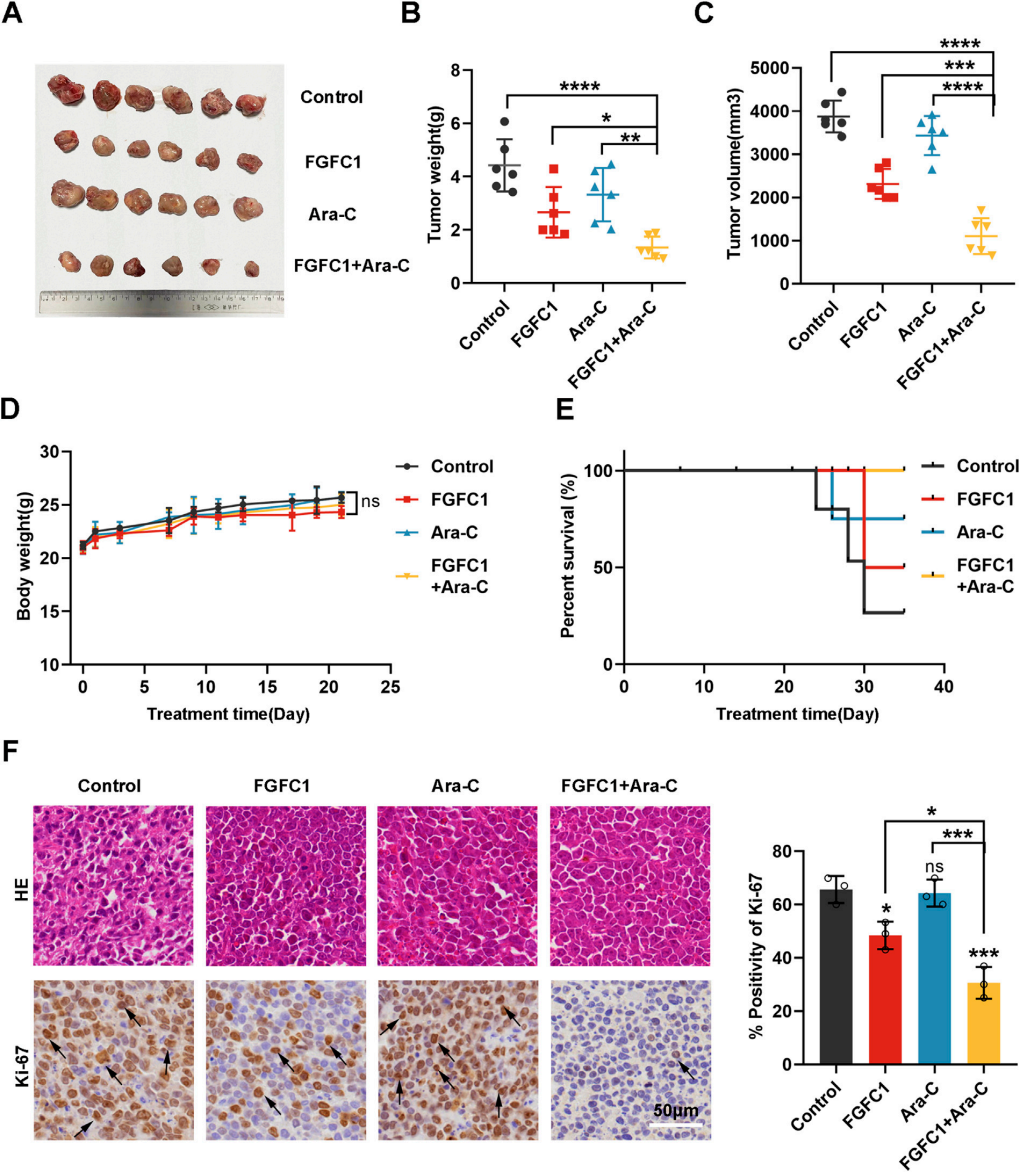
FGFC1 effectively overcomes Ara-C resistance in AML *in vivo*. **(A)** Images of NOG/SCID mice treated with DMSO (2%, ip, n = 6), FGFC1 (30 mg/kg, ip, n = 6), Ara-C (30 mg/kg, ip, n = 6), and FGFC1+ Ara-C (30 mg/kg+30 mg/kg, ip, n = 6) for 3 weeks and then exfoliated to solid images of tumors. **(B,C)** Scatter plots summarizing tumor weight and volume. **(D)** Measured every 2 days, the weight of mice in each group. **(E)** Kaplan-Meier analysis of mouse survival. **(F)** HE staining of tumor tissues, immunocytochemical staining of Ki-67 protein expression in tumor tissues (magnification: ×200, scale bar: 50 μm), and statistical results. All data were shown as mean ± SD, Student’s t-test, *p < 0.05, **p < 0.01, ***p < 0.001, ****p < 0.0001.

To evaluate whether FGFC1 can overcome AML Ara-C resistance *in vivo*, we employed a human tumor cell xenograft model. Ara-C-resistant K562-R cells were injected subcutaneously into the axilla of female NOG/SCID nude mice. After 7–10 days, when the tumor volume reached approximately 100 mm^3^, the mice were administered intraperitoneal injections of DMSO, FGFC1 (30 mg/kg), Ara-C (30 mg/kg), or a combination of FGFC1 and Ara-C for 21 days. Tumor volume and body weight were monitored every 2 days. The results demonstrated that the combination of FGFC1 and Ara-C significantly reduced tumor weight and volume compared to the control, Ara-C monotherapy, and FGFC1 monotherapy groups, indicating that FGFC1 effectively inhibits the growth of AML Ara-C-resistant tumors (Figures 2A–C). Throughout the 21-day treatment period, there were no significant differences in body weight among the control, Ara-C monotherapy, FGFC1 monotherapy, and combination therapy groups. Furthermore, no mice treated with the combination of FGFC1 and Ara-C died, resulting in a higher survival rate compared to the other treatment groups. This partly suggests the safe use of the combination therapy *in vivo* (Figures 2D,E). Immunohistochemical analysis revealed no significant difference in the expression of the proliferation marker Ki-67 between the Ara-C monotherapy group and the control group. However, the expression of Ki-67 was decreased in both the FGFC1 monotherapy group and the combination treatment group compared to the control group. Moreover, the combination treatment of FGFC1 and Ara-C resulted in a more substantial reduction in Ki-67 expression in K562-R xenograft tumors than either monotherapy (Figure 2F). Our results indicated that FGFC1 effectively overcomes AML Ara-C resistance, inhibiting tumor growth. These findings were consistent with our *in vitro* observations of FGFC1-treated AML Ara-C-resistant cells and support the potential of FGFC1 as a novel strategy for treating AML Ara-C resistance.

### 3.3 FGFC1 promotes Ara-C sensitivity in Ara-C-resistant AML cells through apoptosis and pyroptosis

**FIGURE 3:**
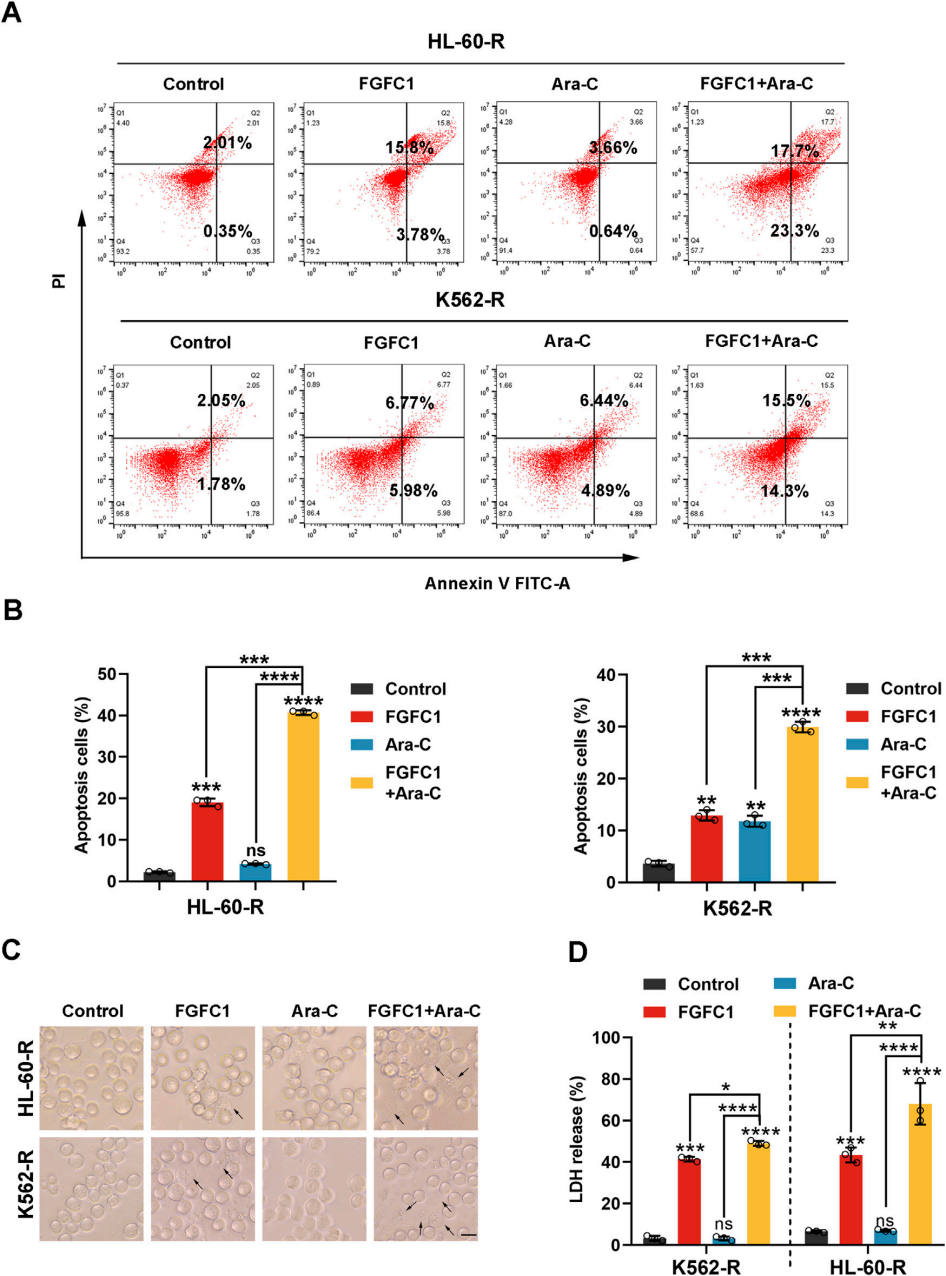
FGFC1 enhances Ara-C responsiveness in Ara-C-resistant AML cells by inducing apoptosis and pyroptosis. **(A,B)** HL-60-R and K562-R cells were exposed to FGFC1 (25 μM) and Ara-C (HL-60-R; 0.3 μM, K562-R; 1.5 μM), alone or in combination for 48 h, followed by Annexin V-FITC/PI staining and flow cytometry analysis. **(C)** Representative bright field microscopy images of the same treated HL-60-R and K562-R cells as in **(A)** for 24 h. The black arrowheads indicated the characteristic balloons on the cell membrane (Scale bar: 50 μm). **(D)** The same treated HL-60-R and K562-R cells, as in Figure 3, were treated for 24 h, after which LDH release was determined. All data were shown as mean ± SD, n = 3, Student’s t-test, *p < 0.05, **p < 0.01, ***p < 0.001, ****p < 0.0001.

Apoptosis and pyroptosis are two forms of programmed cell death that play crucial roles in regulating cell survival and death (Ren et al., 2022). Tumor cells often evade these cell death mechanisms, leading to resistance against therapeutic drugs (Pan et al., 2022). Consequently, to determine whether FGFC1 exerts its inhibitory effects on AML Ara-C-resistant cells by inducing apoptosis and pyroptosis. We analyzed the percentage of HL-60-R and K562-R cells undergoing apoptosis after treatment with FGFC1, Ara-C, and their combination using flow cytometry. After 48 h of treatment with FGFC1 alone and in combination with Ara-C, the percentage of apoptotic cells in HL-60-R and K562-R cells increased compared to the control group. Notably, the combination treatment significantly increased the proportion of apoptotic cells more than FGFC1 alone, indicating that FGFC1 combined with Ara-C can effectively induce apoptosis in AML Ara-C-resistant cells (Figures 3A,B). The differentiation of myeloid cells, a key component of the immune system, is disrupted in AML (Riether et al., 2015). Recent studies suggest that pyroptosis may play a role in promoting myeloid cell differentiation (Solier et al., 2017). Additionally, our previous research demonstrated that FGFC1 regulates the NF-κB pathway, which is associated with immune-inflammatory responses (Feng et al., 2022). Notably, pyroptosis is also influenced by the NF-κB signaling pathway, suggesting a potential link between these processes (Zhang et al., 2024b). Therefore, we further investigated whether FGFC1 induces pyroptosis in AML Ara-C-resistant cells. As shown in Figure 3C, in the combination treatment group, we observed typical features of pyroptosis, including cell swelling and large bubbles escaping from the cell membrane. Given that the disruption of the plasma membrane can lead to the release of cytoplasmic components, we measured LDH release, an indicator of pyroptosis. The results showed no significant change in LDH levels in the Ara-C monotherapy group compared to the control group. However, both the FGFC1 monotherapy and the combination treatment groups significantly promoted LDH release from AML Ara-C-resistant cells. Moreover, the combination treatment significantly increased LDH release compared to either monotherapy (Figure 3D). These findings suggest that FGFC1 combined with Ara-C significantly induces pyroptosis in AML Ara-C-resistant cells.

### 3.4 FGFC1 induces Ara-C-resistant AML cell pyroptosis by triggering cleavage of GSDME

**FIGURE 4:**
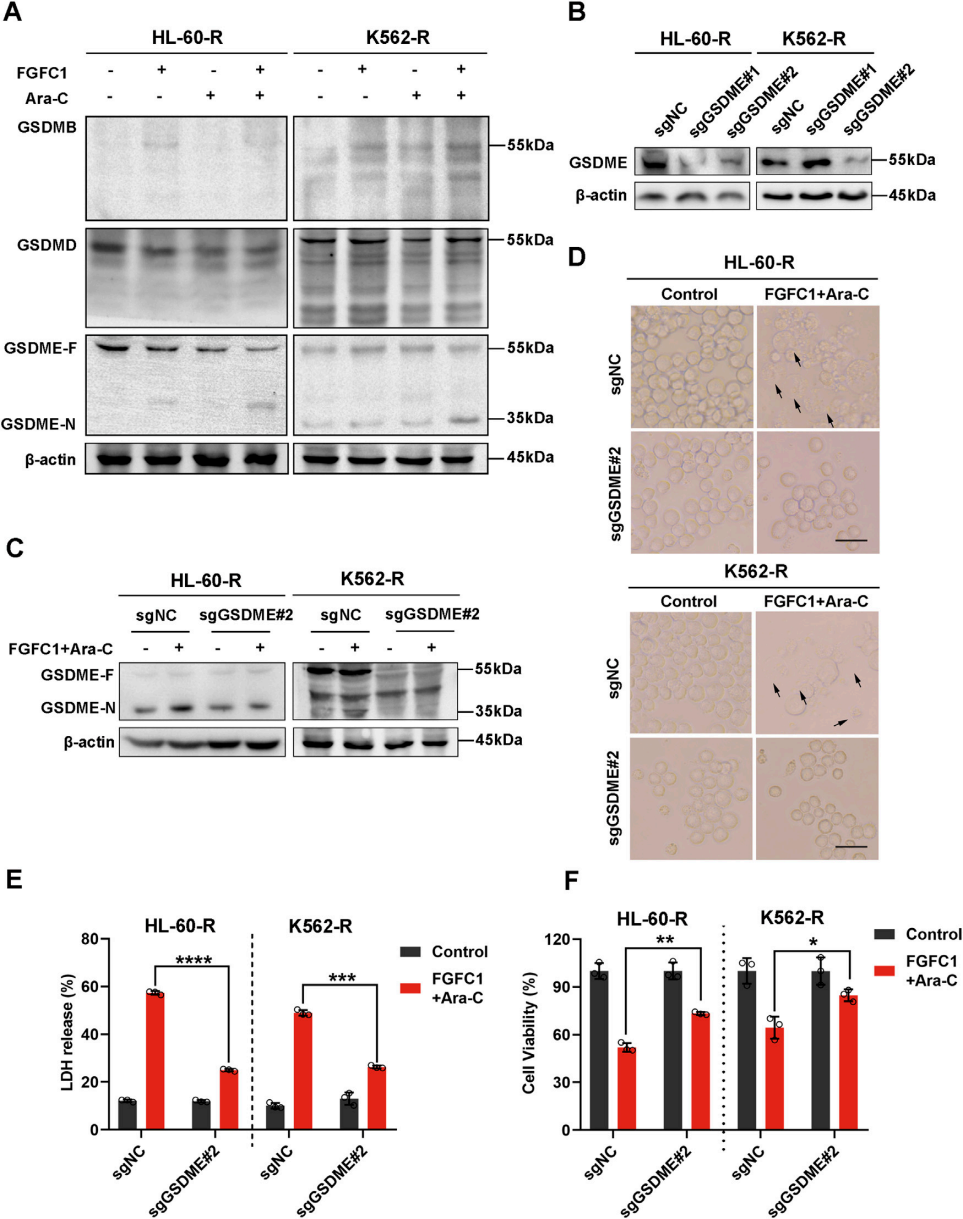
GSDME is essential for FGFC1-induced pyroptosis in Ara-C-resistant AML cells. **(A)** HL-60-R and K562-R cells were treated with FGFC1 (25 μM) and Ara-C (HL-60-R; 0.3 μM, K562-R; 1.5 μM), alone or in combination for 24 h, followed by Western blot analysis to confirm GSDMs protein expression and cleavage. **(B)** Western blot analysis of the GSDME from HL-60-R and K562-R cells with GSDME knockout using CRISPR/Cas 9 method. Parental HL-60-R and K562-R cells were used as the controls. **(C)** Western blot analysis of the GSDME and GSDME-N in the cells from GSDME knockout and non-knockout, and then exposed with the combination of FGFC1 and Ara-C for 24 h. **(D)** Representative bright field microscopy images of the same treated HL-60-R and K562-R cells as in **(C)**. The black arrowheads indicated the characteristic balloons on the cell membrane (Scale bar: 50 μm). **(E,F)** In the same treated HL-60-R and K562-R cells, as in **(C)**, LDH release and cell activity were detected. All data were shown as mean ± SD, n = 3, Student’s t-test, *p < 0.05, **p < 0.01, ***p < 0.001, ****p < 0.0001.

Cellular pyroptosis is dependent on the cleavage and activation of GSDM proteins (Broz et al., 2020). Fanghua Ye et al. examined the expression profiles of GSDMs in AML cell lines and found that GSDMB, GSDMD, and GSDME were expressed in these cells (Ye et al., 2023). Thus, we next determined the expression and cleavage of GSDM proteins following FGFC1 or Ara-C treatment. Our results indicated that the combination of FGFC1 and Ara-C downregulated the expression of full-length GSDME protein (GSDME-F) and upregulated the expression of the GSDME N-terminal fragment (GSDME-N) without affecting the expression of GSDMB and GSDMD proteins in the FGFC1 and Ara-C co-treatment group compared to the control group. This finding suggests that FGFC1 induces pyroptosis in AML cells resistant to Ara-C by cleaving GSDME (Figure 4A). To further demonstrate that GSDME is the executioner protein for FGFC1-induced pyroptosis in Ara-C-resistant AML cells, we utilized the CRISPR-Cas9 system to knock down GSDME in AML Ara-C-resistant HL-60-R and K562-R cell lines (Figure 4B). As shown in Figures 4C–E, GSDME knockdown significantly reduced GSDME cleavage induced by the combination of FGFC1 and Ara-C, leading to decreased plasma membrane bubbling and reduced lactate LDH release in Ara-C-resistant cells. Notably, GSDME knockdown partially reversed the inhibition of cell activity caused by FGFC1 and Ara-C co-treatment (Figure 4F). In conclusion, our results suggest that knocking down GSDME will curb the inhibitory effect of FGFC1 and Ara-C combined treatment. Moreover, the combination of FGFC1 and Ara-C treatment enhances Ara-C sensitivity through GSDME-medicated pyroptosis in AML Ara-C resistance cells.

### 3.5 Activation of mitochondrial pathway and Caspase 3 are involved in AML Ara-C-resistant pyroptosis induced by FGFC1

**FIGURE 5:**
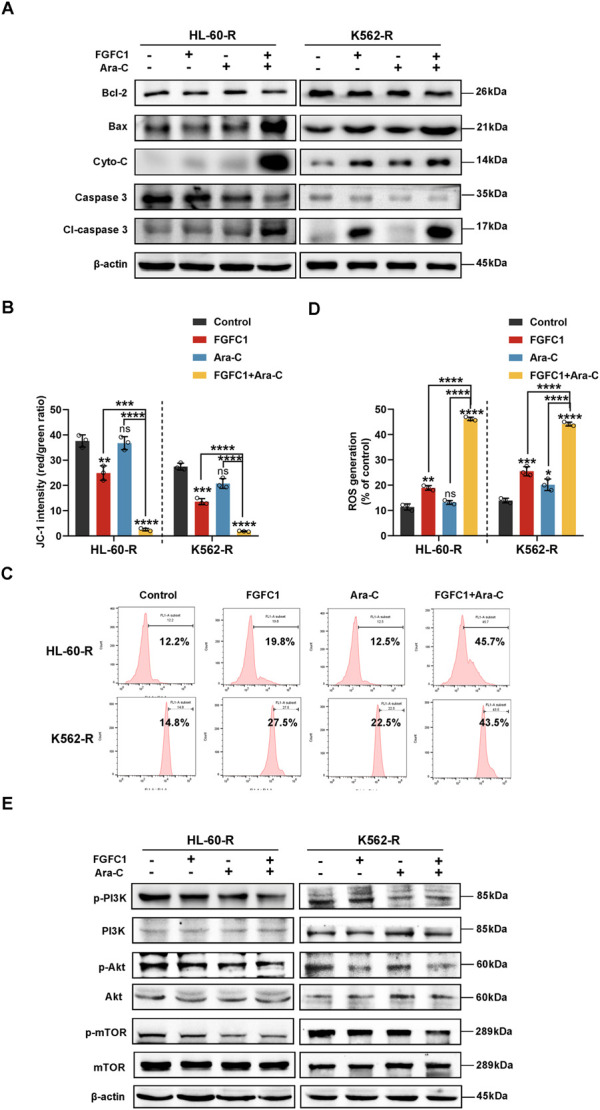
FGFC1 induces mitochondrial dysfunction and ROS accumulation via inhibiting the PI3K/Akt/mTOR pathway in Ara-C-resistant AML cells. **(A)** HL-60-R and K562-R cells were treated with FGFC1(25 μM) and Ara-C (HL-60-R; 0.3 μM, K562-R; 1.5 μM), alone or in combination for 24 h, followed by Western blot analysis to confirm Bcl-2, Bax, Cyto-C, Caspase 3, Cl-Caspase 3 protein expression. **(B)** JC-1 was used to detect MMP changes after 24 h treatment with drugs in **(A)** above. **(C)** After 24 h treatment with drugs in **(A)** above, the ROS level was detected, and the typical flow cytometry histogram showed the fluorescence level of DCFH-Da in cells. **(D)** ROS levels **(C)** statistical plot. **(E)** Western blot analyses of p-PI3K, PI3K, p-Akt, Akt, p-mTOR, mTOR protein expression levels in the same treated HL-60-R and K562-R cells as in **(A)** for 24 h. All data were shown as mean ± SD, n = 3, Student’s t-test, *p < 0.05, **p < 0.01, ***p < 0.001, ****p < 0.0001.

Considering that the intrinsic mitochondrial apoptotic pathway mediates pyroptosis, with both processes occurring simultaneously, we examined the expression of proteins involved in this pathway (Lu et al., 2018). After 24 h of treatment with FGFC1, Ara-C, or their combination, Western blot analysis of HL-60-R and K562-R cells revealed that the combination therapy significantly inhibited the expression of Bcl-2, while promoting the expression of Bax and Cyto-C. Concurrently, there was a significant activation of Caspase 3, which is the key enzyme for GSDME cleavage (Wang et al., 2017). These results suggest that FGFC1 likely induces Caspase 3/GSDME-mediated pyroptosis through the mitochondrial pathway (Figure 5A). Mitochondria play a crucial role in apoptosis, which is thought to proceed via the mitochondria-mediated intrinsic pathway. During this process, increased mitochondrial membrane permeability allows proteins such as Cyto-C to be released into the cytoplasm, triggering the apoptotic cascade (Riether et al., 2015). Given the pivotal role of mitochondria in both apoptosis and pyroptosis, and the fact that FGFC1 promotes Cyto-C release, we further investigated its effect on mitochondrial function in AML cells resistant to Ara-C. Therefore, we assessed the impact of FGFC1 on mitochondrial membrane potential (MMP) using JC-1 staining. As illustrated, compared to the control group and single-drug treatments with FGFC1 or Ara-C, the combination therapy significantly reduced the JC-1 polymer-to-monomer ratio, indicating a decrease in red fluorescence (JC-1 polymer) and an increase in green fluorescence (JC-1 monomer), which suggests FGFC1-induced MMP loss (Figure 5B). In pyroptosis, mitochondria may contribute through the production of reactive oxygen species (ROS) and membrane damage. Additionally, MMP loss is often closely linked to ROS generation (Zhang et al., 2010). Therefore, we further explored the effect of FGFC1 on ROS levels in AML cells resistant to Ara-C (Vaux and Korsmeyer, 1999). The results demonstrated a significant increase in ROS levels in the combination therapy group compared to the single-drug treatments and control group (Figures 5C,D). The histogram results confirmed that the combination of FGFC1 and Ara-C promoted ROS production in Ara-C-resistant AML cells (Supplementary Figure S2). In summary, FGFC1 induces apoptosis and pyroptosis via the mitochondrial pathway in Ara-C-resistant AML cells.

The PI3K/Akt/mTOR signaling pathway is critical for regulating cell growth, proliferation, and differentiation, and plays a significant role in the initiation, progression, treatment, and prognosis of various cancers. Studies have reported that this pathway is activated in leukemia cells, and its excessive activation is associated with Ara-C resistance and relapse in AML (Zhou et al., 2021). Our research team previously demonstrated that FGFC1 efficiently induces mitochondrial dysfunction, and accumulation of ROS by blocking the activation of the PI3K/Akt/mTOR pathway. Therefore, we further investigated the impact of FGFC1 on the PI3K/Akt/mTOR signaling pathway in Ara-C-resistant AML cells. Using Western blot analysis, we evaluated the effects of FGFC1 on this pathway. As shown in Figure 5E, the combination of FGFC1 and Ara-C significantly inhibited the phosphorylation of PI3K, Akt, and mTOR proteins in HL-60-R and K562-R cells compared to single-drug treatments and the control group, while the total protein levels remained unchanged. These results indicate that FGFC1 inhibits the activation of the PI3K/Akt/mTOR signaling pathway in Ara-C-resistant AML cells, thereby reducing their activity. In conclusion, FGFC1 induces apoptosis and GSDME-mediated pyroptosis by inhibiting the PI3K/Akt/mTOR pathway, leading to Caspase 3 cleavage, ultimately overcoming Ara-C resistance in AML.

### 3.6 FGFC1 activates caspase 3/GSDME-induced pyroptosis by inhibiting the PI3K/Akt/mTOR pathway *in vivo*

**FIGURE 6:**
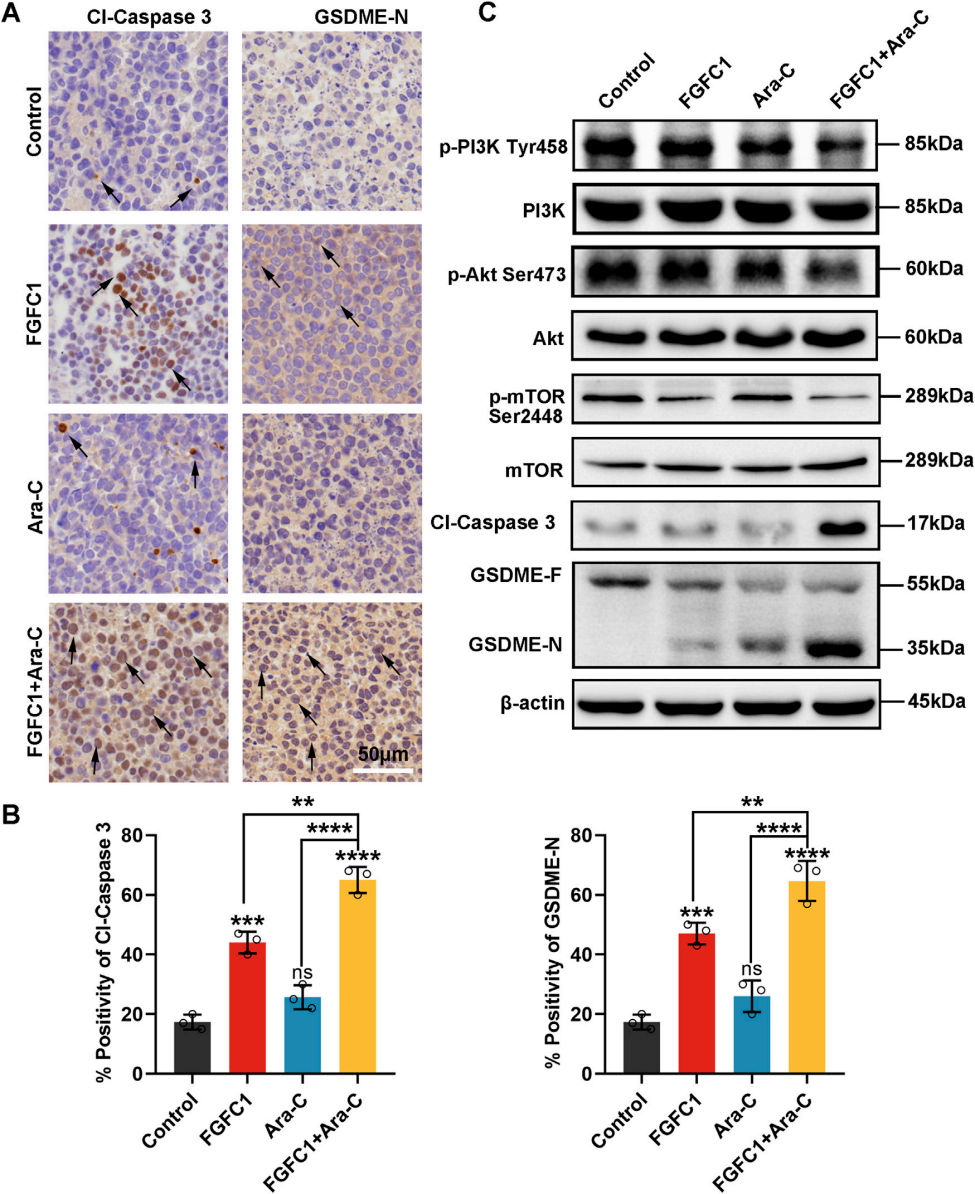
*In vivo*, PI3K/Akt/mTOR pathway is involved in FGFC1 triggers Caspase 3/GSDME-mediated pyroptosis. **(A)** HE staining of tumor tissues, immunocytochemical staining of Cl-caspase, and GSDME-N protein expression in tumor tissues (magnification: ×200, scale bar: 50 μm). **(B)**The IHC results were analyzed by Image-Pro Plus 6.0 (n = 5 fields of view). **(C)** Western blot analysis of p-PI3K, PI3k, p-Akt, Akt, p-mTOR, mTOR, Cl-caspase3, GSDME-F, GSDME-N in tumor tissues, and β-actin was detected as the endogenous loading control, accordingly. All data were shown as mean ± SD, n = 3, Student’s t-test, *p < 0.05, **p < 0.01, ***p < 0.001, ****p < 0.0001.

To investigate the findings *in vitro* that FGFC1 mediates pyroptosis in AML Ara-C-resistant cells through the Caspase 3/GSDME pathway, we established a xenograft NOD/SCID mouse model using AML Ara-C-resistant K562-R cells. Tumor tissues were dissected and subjected to immunohistochemistry analysis (Ye et al., 2023). As shown in Figures 6A,B, the immunohistochemistry results revealed that, compared with the control and single-drug treatment groups, the combined drug treatment group exhibited a significantly larger area of positive staining for Cl-Caspase 3 and GSDME-N proteins in the tumor tissues. This confirmed that FGFC1 mediates pyroptosis in AML Ara-C-resistant cells through the Caspase 3/GSDME pathway *in vivo*. Additionally, Western blot analysis was performed on tumor tissues derived from the xenograft model to examine the expression of PI3K/Akt/mTOR and Caspase 3/GSDME pathway proteins, aiming to determine whether FGFC1 induces pyroptosis and overcomes AML Ara-C resistance through the PI3K/Akt/mTOR signaling pathway (Jiang, 2019). The results demonstrated that the combination of FGFC1 and Ara-C inhibited the phosphorylation of PI3K, Akt, and mTOR proteins in AML Ara-C-resistant cells, without affecting total protein levels. Moreover, Caspase 3 and GSDME were significantly activated, consistent with findings *in vitro* (Figure 6C) (Wu et al., 2024). In conclusion, FGFC1 inhibits the PI3K/Akt/mTOR pathway, activating Caspase 3/GSDME to induce pyroptosis in AML Ara-C-resistant cells.

## 4 Discussion

**FIGURE 7:**
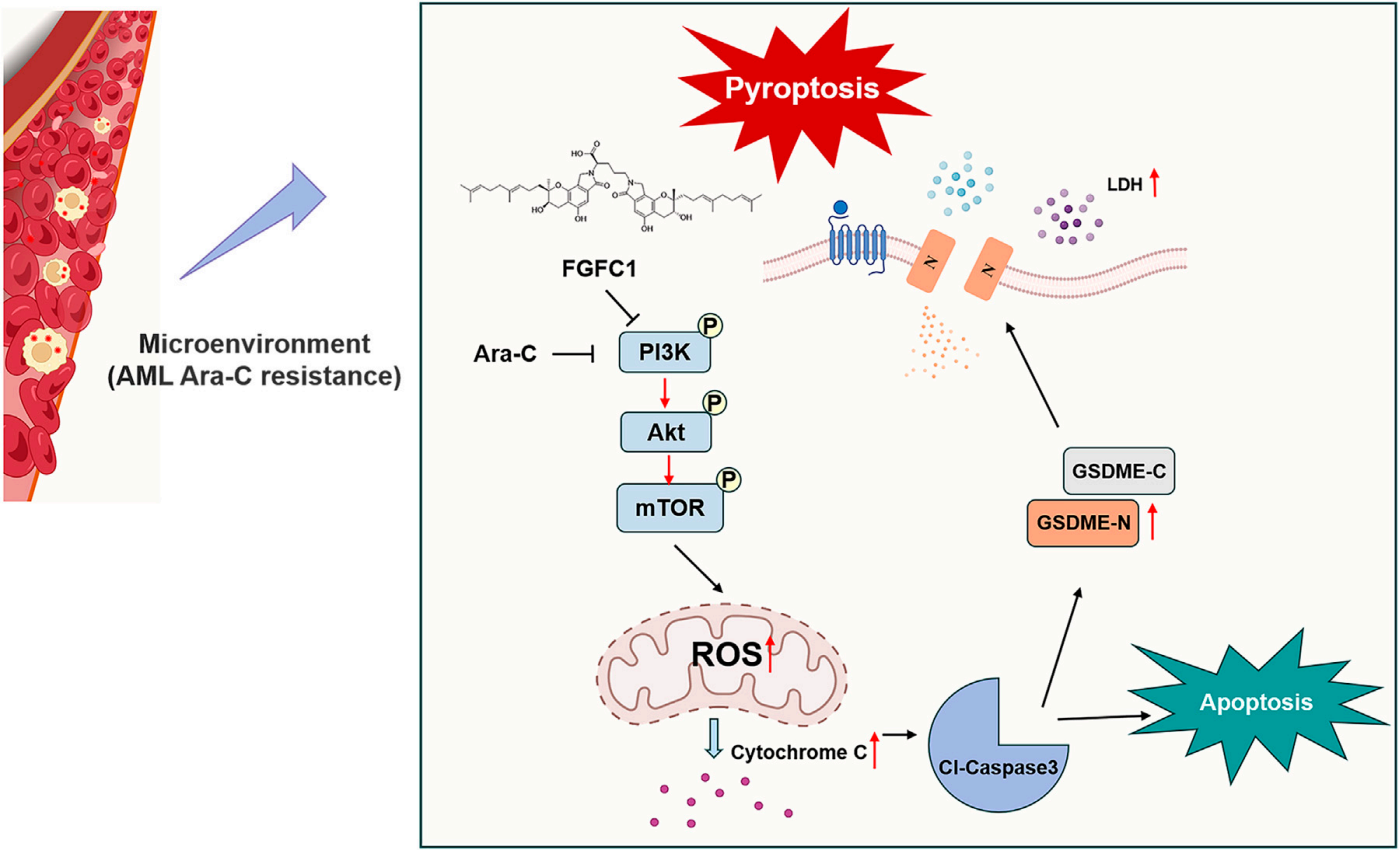
Schematic diagram of FGFC1-induced cell apoptosis and pyroptosis overcoming AML Ara-C resistance.

Although most patients with AML achieve complete remission following conventional chemotherapy, 60%–65% of young adult patients experience drug-resistant relapse within 3 years of diagnosis. Drug-resistant relapse has become a major cause of treatment failure in AML (Stief et al., 2020). As shown in Figure 7, we found that the marine-derived small molecule compound FGFC1 effectively overcame Ara-C resistance in AML. Further mechanistic studies revealed that FGFC1 overcame Ara-C resistance by inhibiting the PI3K/Akt/mTOR signaling pathway, which led to the release of Cyto-C. Consequently, triggered the activation of Caspase-3 and induced apoptosis, as well as GSDME-mediated pyroptosis.

Marine fungi are increasingly recognized for their ability to produce a vast array of bioactive secondary metabolites with unique chemical structures and therapeutic potential. Among these, marine alkaloids, including bisindole alkaloids like FGFC1, are particularly notable for their anticancer properties (Shikov et al., 2020). Our prior research demonstrated the effect of FGFC1 to reverse drug resistance in erlotinib-resistant non-small cell lung cancer and regulate the NF-κB signaling pathway which plays a well-known role in regulating immune response and inflammation, highlighting its therapeutic promise (Feng et al., 2022; Yan et al., 2022). In this study, we established the efficacy of FGFC1 in overcoming Ara-C resistance in AML both *in vitro* and *in vivo*, thereby proposing FGFC1 as a novel therapeutic candidate for resistant AML cases (Figures 1, 2).

To further explore the mechanism by which FGFC1 overcomes Ara-C resistance, we employed flow cytometry, light microscopy, and other methods to investigate how FGFC1 inhibits the growth of Ara-C-resistant cells. We observed that FGFC1 induces both apoptosis and pyroptosis. Pyroptosis, a form of programmed cell death associated with immune and inflammatory responses, is characterized by cell swelling and plasma membrane rupture. Traditionally linked to Caspases 1, 4, 5, and 11 cleaving Gasdermin D (GSDMD) (Yu et al., 2021), recent studies have revealed additional pathways. Chemotherapeutic agents can induce pyroptosis via Caspase-3-mediated cleavage of GSDME, while granzyme B directly cleaves GSDME, and granzyme A hydrolyzes GSDMB (Wang et al., 2017). Our data confirm that FGFC1 activates GSDME through Caspase-3, mediating pyroptosis in Ara-C-resistant AML cells both *in vitro* and *in vivo*. Interestingly, the role of Gasdermin B (GSDMB) in FGFC1’s mechanism remains poorly understood. Although GSDME has been established as a pivotal player, future investigations should explore whether GSDMB also contributes to FGFC1-induced pyroptosis or resistance mechanisms in AML. This knowledge gap underscores the need for further studies to clarify the interplay among Gasdermin family members in this context.

While our current findings demonstrate that FGFC1 triggers caspase-3 activation primarily through mitochondrial dysfunction and ROS accumulation, we cannot exclude the potential involvement of ER stress pathways. endoplasmic reticulum (ER) stress-mediated apoptosis, driven by unfolded protein response (UPR) sensors such as IRE1α, PERK, and ATF6, can activate caspase-3 via CHOP and JNK signaling (Rana, 2020). Future studies will explicitly investigate whether FGFC1 induces ER stress markers (e.g., GRP78, CHOP, XBP1 splicing) and evaluate its contribution to caspase-3 activation in Ara-C-resistant AML cells. Mitochondria play central roles in signal transduction, biosynthesis, and bioenergetics, regulating ROS production and initiating apoptosis. During apoptosis induced by external stimuli, increased mitochondrial membrane permeability leads to a reduction in mitochondrial membrane potential, followed by the release of apoptotic proteins from the mitochondria into the cytoplasm, which ultimately activates the caspase-dependent apoptotic pathway (Estaquier et al., 2012). Additionally, ROS are known to cause cellular damage, and there is a well-established link between mitochondrial dysfunction and ROS production (Ye et al., 2023). Therefore, we examined the effects of FGFC1 on mitochondrial dysfunction and ROS levels. The results demonstrated that FGFC1 significantly decreased MMP and ROS accumulation, leading to the release of Cyto-C, which subsequently activated downstream Caspase 3 to cleave GSDME, thereby inducing both apoptosis and pyroptosis in AML Ara-C-resistant cells. While these findings provide crucial insights, the detailed molecular interactions between FGFC1 and mitochondrial components require further elucidation. Additionally, the off-target effects of FGFC1 and its long-term impact on normal hematopoietic cells must be rigorously assessed to establish its therapeutic safety profile.

The PI3K/AKT/mTOR signaling pathway is a highly conserved network that regulates cell survival, growth, and cycle progression. It is the most frequently activated pathway in human tumors and is often associated with resistance to cancer therapies. Overactivation of PI3K, loss of PTEN function, and mutations in components of the pathway are critical drivers of tumor resistance to therapy and disease progression (Glaviano et al., 2023). Notably, the PI3K/AKT/mTOR pathway is a central regulator of hematopoietic cell proliferation, differentiation, and survival, and is activated in approximately 60% of AML patients, correlating with poor prognosis (Nepstad et al., 2020). Previous studies have shown that FGFC1 inhibits the PI3K/Akt/mTOR and NF-κB pathways in non-small-cell lung cancer (Feng et al., 2022; Yan et al., 2022). Therefore, we investigated the effect of FGFC1 on the PI3K/Akt/mTOR signaling pathway in AML Ara-C-resistant cells. We found that FGFC1 effectively inhibited the activation of the PI3K/Akt/mTOR pathway both *in vitro* and *in vivo*. What’s more, a recent study has revealed that metformin can trigger Caspase3/GSDME-mediated pyroptosis of cancer cells by activating the AMPK/SIRT1/NF-κB pathway (Zheng et al., 2020). Therefore, our study will focus on the role of immune-inflammatory pathways in FGFC1-induced pyroptosis and its reversal of Ara-C resistance in future studies.

In summary, our study demonstrates that the marine-derived small molecule FGFC1 significantly overcomes AML Ara-C resistance. Mechanistically, FGFC1 inhibits the PI3K/Akt/mTOR pathway, leading to mitochondrial dysfunction, Cyto-C release, elevated ROS levels, Caspase 3 activation, and subsequent induction of apoptosis and GSDME-mediated pyroptosis. Overall, our findings suggest that FGFC1 holds promise as a candidate for overcoming AML Ara-C resistance, offering a novel therapeutic strategy for AML patients resistant to Ara-C treatment.

## Data Availability

The raw data supporting the conclusions of this article will be made available by the authors, without undue reservation.

## References

[B1] Bertheloot D. Latz E. Franklin B. S. (2021). Necroptosis, pyroptosis and apoptosis: an intricate game of cell death. Cell. and Mol. Immunol. 18 (5), 1106–1121. 10.1038/s41423-020-00630-3 33785842 PMC8008022

[B2] Broz P. Pelegrín P. Shao F. (2020). The gasdermins, a protein family executing cell death and inflammation. Nat. Rev. Immunol. 20 (3), 143–157. 10.1038/s41577-019-0228-2 31690840

[B3] Cheng M.-Z. Yang B. B. Zhan Z. T. Lin S. M. Fang Z. P. Gao Y. (2023). MACC1 and Gasdermin-E (GSDME) regulate the resistance of colorectal cancer cells to irinotecan. Biochem. Biophysical Res. Commun. 671, 236–245. 10.1016/j.bbrc.2023.06.002 37307707

[B4] Dai J. Qu T. Yin D. Cui Y. Zhang C. Zhang E. (2023). LncRNA LINC00969 promotes acquired gefitinib resistance by epigenetically suppressing of NLRP3 at transcriptional and posttranscriptional levels to inhibit pyroptosis in lung cancer. Cell. Death and Dis. 14 (5), 312. 10.1038/s41419-023-05840-x PMC10167249 37156816

[B5] De Kouchkovsky I. Abdul-Hay M. (2016). Acute myeloid leukemia: a comprehensive review and 2016 update. Blood Cancer J. 6 (7), e441. 10.1038/bcj.2016.50 27367478 PMC5030376

[B6] Estaquier J. Vallette F. Vayssiere J. L. Mignotte B. (2012). ‘The mitochondrial pathways of apoptosis’, in Scatena R. Bottoni P. Giardina B. (eds) Advances in mitochondrial medicine. Dordrecht: Springer Netherlands (Advances in Experimental Medicine and Biology), pp. 157–183. 10.1007/978-94-007-2869-1_7 22399422

[B7] Feng J. Li S. Zhang B. Duan N. Zhou R. Yan S. (2022). FGFC1 exhibits anti-cancer activity via inhibiting NF-κB signaling pathway in EGFR-mutant NSCLC cells. Mar. Drugs 20 (1), 76. 10.3390/md20010076 35049931 PMC8781927

[B8] Forsythe A. Sandman K. (2021). What does the economic burden of acute myeloid leukemia treatment look like for the next decade? An analysis of key findings, challenges and recommendations. J. Blood Med. 12, 245–255. 10.2147/JBM.S279736 33981169 PMC8107010

[B9] Glaviano A. Foo A. S. C. Lam H. Y. Yap K. C. H. Jacot W. Jones R. H. (2023). PI3K/AKT/mTOR signaling transduction pathway and targeted therapies in cancer. Mol. Cancer 22 (1), 138. 10.1186/s12943-023-01827-6 37596643 PMC10436543

[B10] Haque N. Parveen S. Tang T. Wei J. Huang Z. (2022). Marine natural products in clinical use. Mar. Drugs 20 (8), 528. 10.3390/md20080528 36005531 PMC9410185

[B11] Jia Y. Wang X. Deng Y. Li S. Xu X. Qin Y. (2023). Pyroptosis provides new strategies for the treatment of cancer. J. Cancer 14 (1), 140–151. 10.7150/jca.77965 36605484 PMC9809330

[B12] Jiang L. Wang P. Sun Y. J. Wu Y. J. (2019). Ivermectin reverses the drug resistance in cancer cells through EGFR/ERK/Akt/NF-κB pathway. Clin. Cancer Res. 38, 265. 10.1186/s13046-019-1251-7 PMC6580523 31215501

[B13] Li S. Yue M. Xu H. Zhang X. Mao T. Quan M. (2023). Chemotherapeutic drugs-induced pyroptosis mediated by gasdermin E promotes the progression and chemoresistance of pancreatic cancer. Cancer Lett. 564, 216206. 10.1016/j.canlet.2023.216206 37120007

[B14] Liu H. (2021). Emerging agents and regimens for AML. J. Hematol. and Oncol. 14 (1), 49. 10.1186/s13045-021-01062-w 33757574 PMC7989091

[B15] Lu H. Zhang S. Wu J. Chen M. Cai M. C. Fu Y. (2018). Molecular targeted therapies elicit concurrent apoptotic and GSDME-dependent pyroptotic tumor cell death. Clin. Cancer Res. 24 (23), 6066–6077. 10.1158/1078-0432.CCR-18-1478 30061362

[B16] Lu X. Yang F. Chen D. Zhao Q. Chen D. Ping H. (2020). Quercetin reverses docetaxel resistance in prostate cancer *via* androgen receptor and PI3K/Akt signaling pathways. Int. J. Biol. Sci. 16 (7), 1121–1134. 10.7150/ijbs.41686 32174789 PMC7053318

[B17] Nepstad I. Hatfield K. J. Grønningsæter I. S. Reikvam H. (2020). The PI3K-Akt-mTOR signaling pathway in human acute myeloid leukemia (AML) cells. Int. J. Mol. Sci. 21 (8), 2907. 10.3390/ijms21082907 32326335 PMC7215987

[B18] Pan J. Jiang Y. Li C. Jin T. Yu K. Jin Z. (2022). Characteristics of pyroptosis-related subtypes and novel scoring tool for the prognosis and chemotherapy response in acute myeloid leukemia. Front. Oncol. 12, 898236. 10.3389/fonc.2022.898236 35756629 PMC9229173

[B19] Rana S. V. S. (2020). Endoplasmic reticulum stress induced by toxic Elements—A review of recent developments. Biol. Trace Elem. Res. 196 (1), 10–19. 10.1007/s12011-019-01903-3 31686395

[B20] Ren X. Xie X. Chen B. Liu L. Jiang C. Qian Q. (2021). Marine natural products: a potential source of anti-hepatocellular carcinoma drugs. J. Med. Chem. 64 (12), 7879–7899. 10.1021/acs.jmedchem.0c02026 34128674

[B21] Ren J. Tao Y. Peng M. Xiao Q. Jing Y. Huang J. (2022). Targeted activation of GPER enhances the efficacy of venetoclax by boosting leukemic pyroptosis and CD8+ T cell immune function in acute myeloid leukemia. Cell. Death and Dis. 13 (10), 915. 10.1038/s41419-022-05357-9 PMC9622865 36316313

[B22] Riether C. Schürch C. M. Ochsenbein A. F. (2015). Regulation of hematopoietic and leukemic stem cells by the immune system. Cell. Death and Differ. 22 (2), 187–198. 10.1038/cdd.2014.89 PMC4291501 24992931

[B23] Shikov A. N. Flisyuk E. V. Obluchinskaya E. D. Pozharitskaya O. N. (2020). Pharmacokinetics of marine-derived drugs. Mar. Drugs 18 (11), 557. 10.3390/md18110557 33182407 PMC7698100

[B24] Solier S. Fontenay M. Vainchenker W. Droin N. Solary E. (2017). Non-apoptotic functions of caspases in myeloid cell differentiation. Cell. Death and Differ. 24 (8), 1337–1347. 10.1038/cdd.2017.19 PMC5520450 28211870

[B25] Stief S. M. Hanneforth A. L. Weser S. Mattes R. Carlet M. Liu W. H. (2020). Loss of KDM6A confers drug resistance in acute myeloid leukemia. Leukemia 34 (1), 50–62. 10.1038/s41375-019-0497-6 31201358 PMC7214274

[B26] Su L. Chen Y. Huang C. Wu S. Wang X. Zhao X. (2023). Targeting src reactivates pyroptosis to reverse chemoresistance in lung and pancreatic cancer models. Sci. Transl. Med. 15 (678), eabl7895. 10.1126/scitranslmed.abl7895 36630483

[B27] Vaux D. L. Korsmeyer S. J. (1999). Cell death in development. Cell. 96 (2), 245–254. 10.1016/S0092-8674(00)80564-4 9988219

[B28] Venugopal S. Sekeres M. A. (2024). Contemporary management of acute myeloid leukemia: a review. JAMA Oncol. 10 (10), 1417–1425. 10.1001/jamaoncol.2024.2662 39115831

[B29] Wang Y. Gao W. Shi X. Ding J. Liu W. He H. (2017). Chemotherapy drugs induce pyroptosis through caspase-3 cleavage of a gasdermin. Nature 547 (7661), 99–103. 10.1038/nature22393 28459430

[B30] Wu W. Wu M. Y. Dai T. Ke L. N. Shi Y. Hu J. (2024). Terphenyllin induces CASP3-dependent apoptosis and pyroptosis in A375 cells through upregulation of p53. Cell. Commun. Signal. 22, 409. 10.1186/s12964-024-01784-7 39169379 PMC11337594

[B31] Yan S. Zhang B. Feng J. Wu H. Duan N. Zhu Y. (2022). FGFC1 selectively inhibits Erlotinib-Resistant non-small cell lung cancer *via* elevation of ROS mediated by the EGFR/PI3K/Akt/mTOR pathway. Front. Pharmacol. 12, 764699. 10.3389/fphar.2021.764699 35126111 PMC8807551

[B32] Yang H. Zhang Q. Zhang B. Zhao Y. Wang N. (2023). Potential active marine peptides as anti-aging drugs or drug candidates. Mar. Drugs 21 (3), 144. 10.3390/md21030144 36976193 PMC10053682

[B33] Yang C. Hu Y. Gao L. Li Z. Zhang Y. Zhuo R. (2024). Anagrelide and idarubicin combination induces GSDME-Mediated pyroptosis as a potential therapy for high-PDE3A acute myeloid leukemia. Leukemia 39, 98–111. 10.1038/s41375-024-02437-x 39406931

[B34] Ye F. Zhang W. Fan C. Dong J. Peng M. Deng W. (2023). Antileukemic effect of venetoclax and hypomethylating agents *via* caspase-3/GSDME-mediated pyroptosis. J. Transl. Med. 21 (1), 606. 10.1186/s12967-023-04481-0 37679782 PMC10486003

[B35] Yu P. Zhang X. Liu N. Tang L. Peng C. Chen X. (2021). Pyroptosis: mechanisms and diseases. Signal Transduct. Target. Ther. 6 (1), 128. 10.1038/s41392-021-00507-5 33776057 PMC8005494

[B36] Zhang Q. Raoof M. Chen Y. Sumi Y. Sursal T. Junger W. (2010). Circulating mitochondrial DAMPs cause inflammatory responses to injury. Nature 464 (7285), 104–107. 10.1038/nature08780 20203610 PMC2843437

[B37] Zhang J. Wang Y. Yin C. Gong P. Zhang Z. Zhao L. (2022). Artesunate improves venetoclax plus cytarabine AML cell targeting by regulating the Noxa/Bim/Mcl-1/p-Chk1 axis. Cell. Death and Dis. 13 (4), 379. 10.1038/s41419-022-04810-z PMC9021233 35443722

[B38] Zhang H. Zhu H. Sheng Y. Cheng Z. Peng H. (2024a). A novel prognostic model based on pyroptosis signature in AML. Heliyon 10 (17), e36624. 10.1016/j.heliyon.2024.e36624 39263179 PMC11387551

[B39] Zhang Z. Yang Z. Wang S. Wang X. Mao J. (2024b). Overview of pyroptosis mechanism and in-depth analysis of cardiomyocyte pyroptosis mediated by NF-κB pathway in heart failure. Biomed. and Pharmacother. 179, 117367. 10.1016/j.biopha.2024.117367 39214011

[B40] Zheng Z. Bian Y. Zhang Y. Ren G. Li G. (2020). Metformin activates AMPK/SIRT1/NF-κB pathway and induces mitochondrial dysfunction to drive caspase3/GSDME-mediated cancer cell pyroptosis. Cell. Cycle 19 (10), 1089–1104. 10.1080/15384101.2020.1743911 32286137 PMC7217368

[B41] Zhou C. Du J. Zhao L. Liu W. Zhao T. Liang H. (2021). GLI1 reduces drug sensitivity by regulating cell cycle through PI3K/AKT/GSK3/CDK pathway in acute myeloid leukemia. Cell. Death and Dis. 12 (3), 231. 10.1038/s41419-021-03504-2 PMC7930050 33658491

