# Supplementary figures and images for: FGFC1 overcomes Ara-C resistance in acute myeloid leukemia by inducing apoptosis and pyroptosis
Source: Front Pharmacol. 2025 Aug 14;16:1584376. doi: 10.3389/fphar.2025.1584376 (PMC12391149; doi:10.3389/fphar.2025.1584376)

# Supplement Data

|       | HL-60-R |   |   |   | K562-R |   |   |   |
|-------|---------|---|---|---|--------|---|---|---|
| FGFC1 | -       | + | - | + | -      | + | - | + |
| Ara-C | -       | - | + | + | -      | - | + | + |

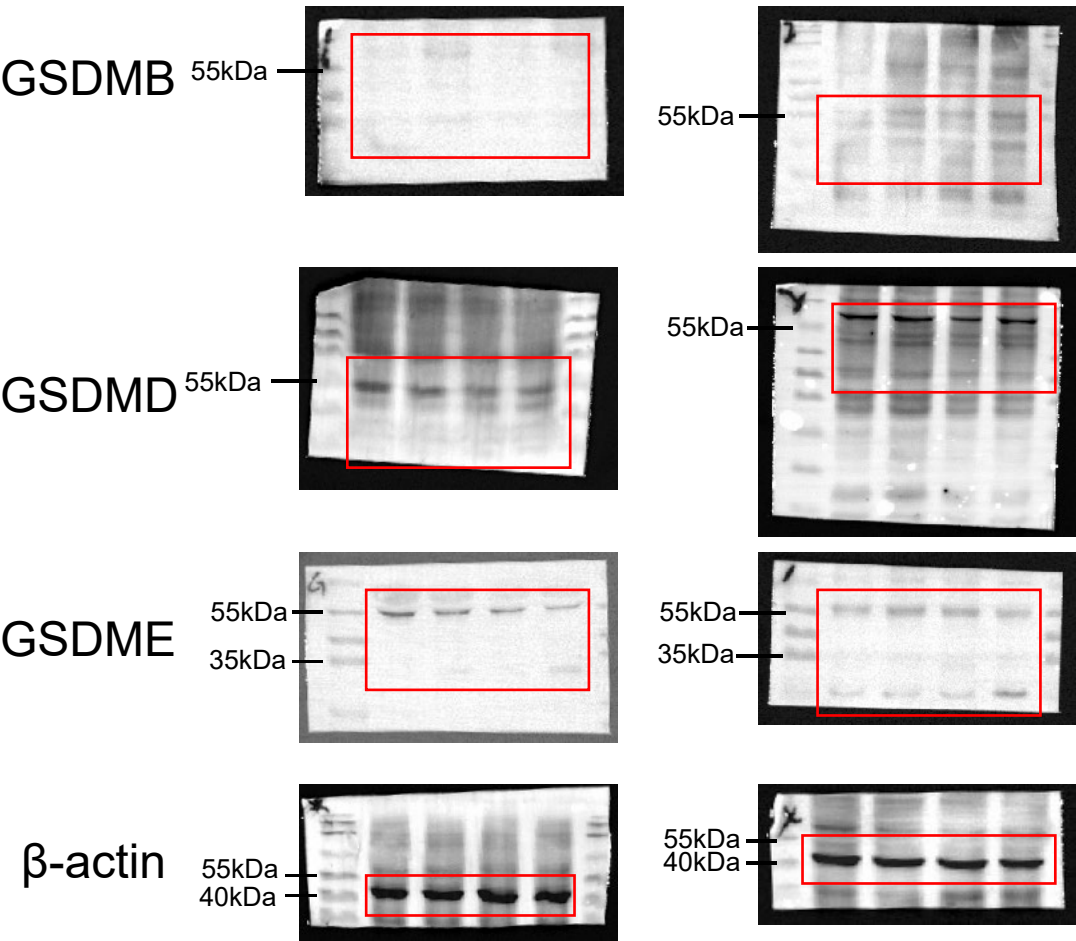

Figure S1

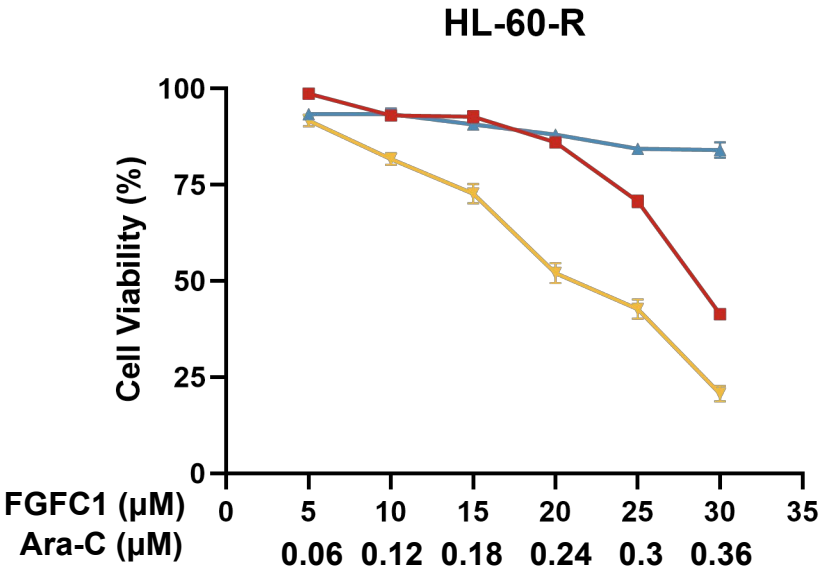

■ FGFC1  
▲ Ara-C  
▼ FGFC1+Ara-C

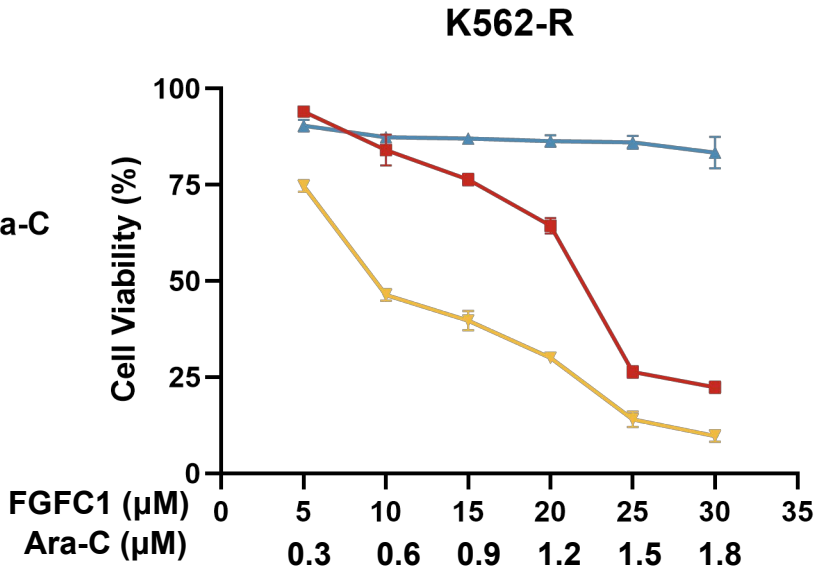

■ FGFC1  
▲ Ara-C  
▼ FGFC1+Ara-C

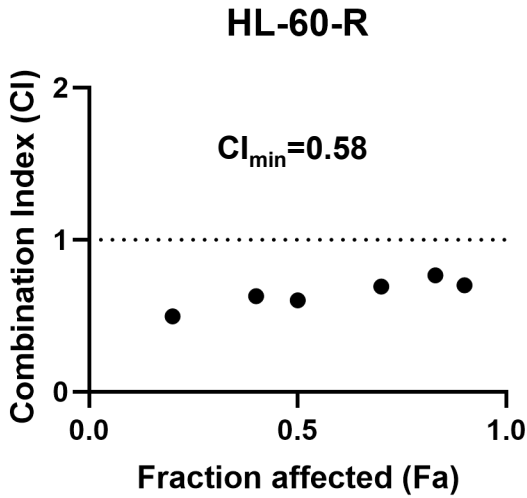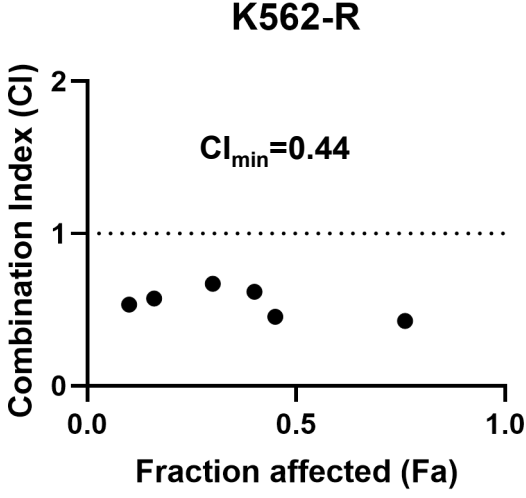

Figure S2

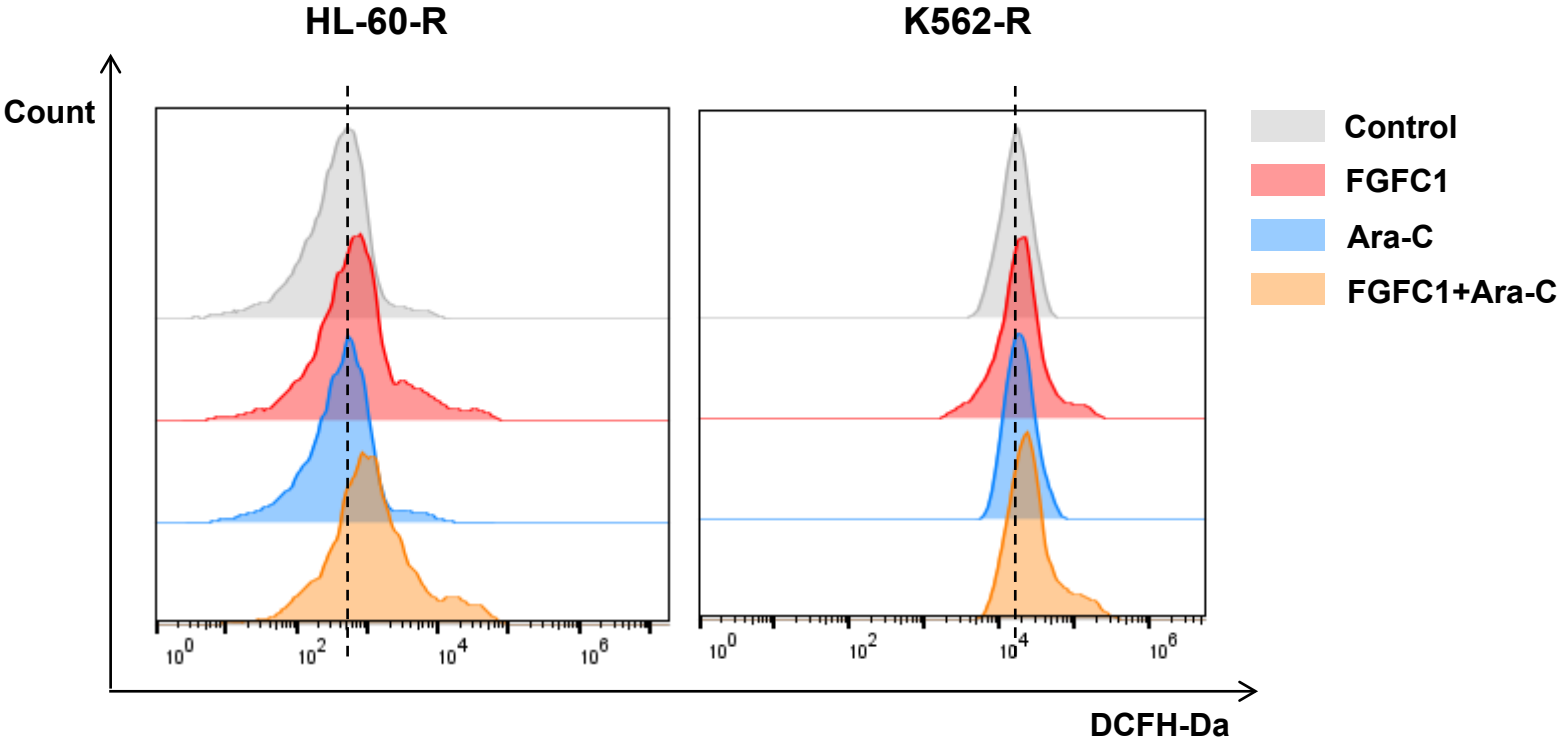

Supplement: Supplementary file 1 [file DataSheet1.pdf]
